# Reduced Local Response to Corticosteroids in Eosinophilic Chronic Rhinosinusitis with Asthma

**DOI:** 10.3390/biom10020326

**Published:** 2020-02-18

**Authors:** Yoshiki Kobayashi, Akira Kanda, Yasutaka Yun, Dan Van Bui, Kensuke Suzuki, Shunsuke Sawada, Mikiya Asako, Hiroshi Iwai

**Affiliations:** 1 Airway Disease Section, Department of Otorhinolaryngology, Kansai Medical University, Hirakata, Osaka 573-1010, Japanyunys@hirakata.kmu.ac.jp (Y.Y.);; 2 Allergic Center, Kansai Medical University Hospital, Hirakata, Osaka 573-1010, Japan

**Keywords:** asthma, corticosteroid sensitivity, eosinophilic chronic rhinosinusitis, glucocorticoid receptor, protein phosphatase 2A

## Abstract

Eosinophilic chronic rhinosinusitis (ECRS), a subgroup of chronic rhinosinusitis with nasal polyps, is recognized as a refractory eosinophilic disorder characterized by both upper and lower airway inflammation. In some severe cases, disease control is poor, likely due to local steroid insensitivity. In this study, we focused on protein phosphatase 2A (PP2A), a key factor regulating glucocorticoid receptor (GR) nuclear translocation, and examined its association with local responses to corticosteroids in eosinophilic airway inflammation. Our results indicated reduced responses to corticosteroids in nasal epithelial cells from ECRS patients with asthma, which were also associated with decreased PP2A mRNA expression. Eosinophil peroxidase stimulates elevated PP2A phosphorylation levels, reducing PP2A protein expression and activity. In addition, mRNA levels of inflammatory mediators (TSLP, IL-25, IL-33, CCL4, CCL5, CCL11, and CCL26) associated with eosinophilic airway inflammation in epithelial cells were increased in nasal polyps (eosinophil-rich areas) compared with those in uncinate process tissues (eosinophil-poor areas) from the same patients. PP2A reduction by siRNA reduced GR nuclear translocation, whereas PP2A overexpression by plasmid transfection, or PP2A activation by formoterol, enhanced GR nuclear translocation. Collectively, our findings indicate that PP2A may represent a promising therapeutic target in refractory eosinophilic airway inflammation characterized by local steroid insensitivity.

## 1. Introduction

Eosinophilic chronic rhinosinusitis (ECRS), a subgroup of chronic rhinosinusitis with nasal polyps is characterized by ethmoid-predominant sinusitis with eosinophilic inflammation [1,2]. ECRS is a refractory eosinophilic airway disease because ECRS patients have a much higher incidence (about 50%) of bronchial asthma [3]. In general, conventional ECRS treatments, including intranasal corticosteroids and oral corticosteroids, do not control the disease adequately. Recently, we reported the usefulness of inhaled corticosteroid (ICS) exhalation through the nose (ETN) treatment for ECRS patients with bronchial asthma [4,5,6]. ICS-ETN treatment reduces operative interventions by half [6] and reduces recurrence rates within three years, after endoscopic sinus surgery (ESS), by half in patients with severe ECRS (unpublished data, in preparation). However, about 30% of these patients experienced recurrence even after ESS. Bronchial asthma is a known risk factor of relapse after ESS [7], suggesting that severe ECRS with asthma is much more difficult to control because of reduced corticosteroid sensitivity [8].

Nuclear translocation of the glucocorticoid receptor (GR) is a critical process that mediates corticosteroids’ biological actions [9]. In patients with refractory airway inflammation, including severe asthma, the reduced nuclear translocation of GR is an important molecular mechanisms of corticosteroid resistance. GR-Ser^226^ phosphorylation induces this resistance due to the reduced activity and expression of phosphatases, such as protein phosphatase 2A (PP2A) [10,11,12]. Interestingly, a recent report has shown that PP2A expression is lower in nasal polyps from ECRS patients compared with controls [13]. In addition, there are reports of reduced corticosteroid responses in nasal polyp epithelial cells from patients with refractory chronic rhinosinusitis [8]. However, the local response to corticosteroids in patients with ECRS and the underlying mechanisms have not been elucidated fully.

In this study, we focused on corticosteroid sensitivity in nasal bronchial cells from patients with ECRS and investigated the molecular mechanisms of steroid resistance.

## 2. Materials and Methods 

### 2.1. Cell Preparation

Tissue specimens were obtained from nasal polyps in the ethmoid sinuses of patients with ECRS and uncinate process tissues of patients with allergic rhinitis or ECRS during surgery. Nasal epithelial cells were collected from healthy volunteers and patients with allergic rhinitis, ECRS, or chronic rhinosinusitis (non-ECRS), by brushing the inferior turbinate (middle meatus side) with a cervical cytology brush. The desquamated epithelium from the tissue samples and nasal epithelial cells was incubated until EpCAM (Millipore, Temecula, CA, USA) confirmed a monolayer of epithelial-like cells. The human bronchial epithelial cell line BEAS-2B was obtained from the European Collection of Authenticated Cell Culture (Salisbury, UK). Eosinophils (purity > 98%) were isolated from the peripheral blood of healthy volunteers with mild eosinophilia (approximately 4%–8% of total white blood cells) by negative selection using a MACS system in conjunction with the Eosinophil Isolation Kit (Miltenyi Biotec, Bergisch Gladbach, Germany). Table S1 shows the characteristics of subjects. This study was approved by the local ethics committee of Kansai Medical University (approval number: KanIRin1313), and written informed consent was obtained from each patient or volunteer.

### 2.2. Corticosteroid Sensitivity

Nasal epithelial cells were treated with dexamethasone for 45 min, followed by TNFα (10 ng/mL) stimulation overnight. The ability of dexamethasone to inhibit TNFα-induced CXCL8 release was determined in cell medium by sandwich ELISA according to the manufacturer’s instructions (R&D Systems, Minneapolis, MN, USA). IC_50_ of dexamethasone on CXCL8 production (Dex-IC_50_), calculated using Prism^®^ 6.0 statistical software (GraphPad, San Diego, CA, USA), was used as a marker for corticosteroid sensitivity. In addition, BEAS-2B cells were treated overnight with FITC-conjugated dexamethasone (Molecular Probes, Eugene, OR; 10^−6^ M). The nuclear fraction was prepared by 10 min incubation with hypotonic buffer (Epigentec, Farmingdale, NY, USA), followed by pulse vortexing. Dexamethasone’s ability to translocate into the nucleus was evaluated by FITC-dexamethasone detected in the nuclei using a fluorescent plate reader.

### 2.3. Quantitative RT-PCR

Total RNA extraction and reverse transcription were performed using a PureLink RNA Micro kit (Invitrogen, Carlsbad, CA, USA) and a PrimeScript RT MasterMix (Perfect Real Time; Takara Bio, Shiga, Japan). Gene transcript levels of protein phosphatase 2 catalytic subunit alpha isozyme (*PPP2CA*), protein tyrosine phosphatase-RR (*PTP-RR*), *TSLP*, *IL-25*, *IL-33*, *CCL4*, *CCL5*, *CCL11*, *CCL26,* and glyceraldehyde 3-phosphate dehydrogenase (*GAPDH*) were quantified by real-time PCR using a Rotor-Gene SYBR Green PCR kit (Qiagen, Hilden, Germany) on a Rotor-Gene Q HRM (Corbett Research, Cambridge, UK). Table S2 shows detail of the amplification primers.

### 2.4. Immunofluorescence Staining

After treatment with dexamethasone (10^−7^ M) for 1 h, the cells were fixed with 4% formaldehyde for 20 min, permeabilized, and blocked. The cells were then incubated with the primary antibodies (mouse monoclonal antibody to GR; Abcam, Cambridge, UK and rabbit polyclonal antibody to PP2A; GeneTex, Alton Pkwy Irvine, CA, USA), followed by the fluorescently labeled secondary antibodies (Alexa 488 donkey anti-mouse and APC donkey anti-rabbit; Jackson Immuno Research, West Grove, PA, USA). Control antibodies and Hoechst (Invitrogen, Paisley, UK) were included in each experiment. GR’s ability to translocate into the nucleus was evaluated using a Carl Zeiss LSM700 confocal microscope.

### 2.5. In-Cell Western Assay

BEAS-2B cells fixed with 4% formaldehyde for 20 min were permeabilized and blocked. Cells were incubated with primary antibodies (mouse monoclonal antibody to phospho-PP2A; Santa Cruz Biotechnology, Dallas, TX and rabbit polyclonal antibody to PP2A; GeneTex) and the fluorescently-labeled secondary antibodies (IRDye 800CW goat anti-mouse and IRDye 680RD goat anti-rabbit; LI-COR Bioscience, Lincoln, NE, USA). Ratio of fluorescence intensity of phospho-PP2A to that of PP2A was analyzed by Odyssey infrared imaging system (LI-COR Bioscience) according to the manufacturer’s instructions.

### 2.6. Cell lysis, Immunoprecipitation, and Western Blotting

Cell protein extracts were prepared using modified RIPA buffer (50 mM Tris HCL pH 7.4, 1.0% NP-40, 0.25% Na-deoxycholate, 150 mM NaCl with freshly added complete protease inhibitor), as described previously [10]. Phosphatase inhibitor was used as needed. Protein concentration was determined using the Bio-Rad Protein Assay (Bio-Rad, Hercules, CA, USA). Immunoprecipitation was conducted with anti-GR antibody (Cell Signaling Technology, Danvers, MA, USA) or anti-PP2A antibody (Santa Cruz Biotechnology). Protein extracts or immunoprecipitates were separated by SDS-PAGE (Bio-Rad) and detected by Western Blot analysis using Odyssey infrared imaging system (LI-COR Bioscience) according to the manufacturer’s instructions. The mouse monoclonal antibody to PP2A and GR (Santa Cruz Biotechnology), the rabbit polyclonal PTP-RR antibody (Aviva Systems Biology, San Diego, CA, USA), and the rabbit monoclonal mucin 1 antibody (Abcam) were used for primary antibodies. β-actin expression was used as a control for protein loading as needed.

### 2.7. Phosphatase Activity

Phosphatase activity in immunopurified PP2A was assayed using SensoLyte^TM^ MFP Protein Phosphatase Assay Kit (AnaSpec, San Jose, CA, USA) as previously described [10].

### 2.8. RNA Interference

PP2ACα siRNAs and non-silencing scrambled control siRNA were purchased from QIAGEN (Crawley, UK). The siRNA sequences (0.6 μM) were transfected using Lipofectamine RNAiMAX Reagent (Invitrogen) according to the manufacturer’s specifications.

### 2.9. Transfection

Transfections were done by Xfect Transfection Reagent (Takara Bio), accoding to the manufacturer’s specifications. A total of 2 μg of DNA/plasmids (Thermo Fisher Scientific, Tokyo, Japan) containing PP2Acα gene were transfected to BEAS-2B cells.

### 2.10. Statistical Analysis

Comparisons of two groups of data were performed using Mann-Whitney U test or paired *t*-test. Correlation coefficients were calculated with the use of Spearman’s rank method. Other data were analyzed by ANOVA with post *hoc* test adjusted for multiple comparisons (Dunn’s test or Newman-Keuls test), as appropriate. Differences were considered statistically significant if *p* value was <0.05. Descriptive statistics were expressed as means ± SEM.

## 3. Results

### 3.1. Local Responses to Corticosteroid are Reduced in ECRS Patients with Asthma

**Figure 1:**
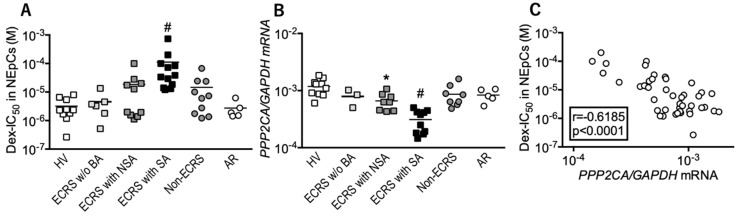
Responses to corticosteroids and protein phosphatase 2A (PP2A) expression in nasal epithelial cells (NEpCs). (**A**) IC_50_ values for dexamethasone on TNFα-induced CXCL8 production (Dex-IC_50_) were measured as a marker of corticosteroid sensitivity. (**B**) PP2A mRNA levels were determined using RT-PCR. (**C**) Correlation between Dex-IC_50_ and PP2A mRNA levels. Individual values of patients in each group are shown. ^#^ *p* < 0.05 (vs. other groups), * *p* < 0.05 (vs. healthy volunteers). HV: healthy volunteers; ECRS: eosinophilic chronic rhinosinusitis; BA: bronchial asthma; NSA: non-severe asthma; SA: severe asthma; AR: allergic rhinitis.

In this study, we evaluated the corticosteroid sensitivity of nasal epithelial cells. Our results indicated that Dex-IC_50_ was significantly elevated in ECRS patients with severe asthma compared with other groups (Figure 1A). PP2A mRNA expression was reduced in ECRS patients with asthma (Figure 1B). PP2A mRNA expression was negatively correlated with Dex-IC_50_ values (Figure 1C), suggesting that ECRS patients with asthma have a reduced local response to corticosteroids.

### 3.2. Decreased PP2A in Eosinophilic Inflammation Leads to Reduced GR Nuclear Translocation

**Figure 2:**
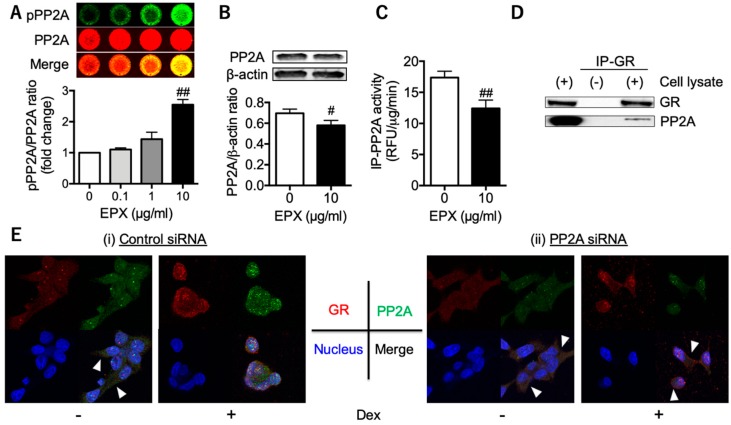
PP2A’s involvement in glucocorticoid receptor (GR) nuclear translocation. Phosphorylation levels of PP2A (**A**), PP2A protein expression (**B**), and activity (**C**) in human bronchial epithelial cells (BEAS-2B cells). BEAS-2B cells were co-incubated with recombinant eosinophil peroxidase (EPX) for 15 min (**A**) or 3 days (**B**,**C**). Data in A are expressed as the fold change relative to the vehicle. Values in A, B, and C represent the mean ± SEM values of four experiments: ^#^ *p* < 0.05, ^##^ *p* < 0.01 (vs. vehicle). (**D**) GR and PP2A expression in whole cell extracts (left lane) and in GR-immunoprecipitates (right lane). (**E**) Dexamethasone (Dex)-induced GR nuclear translocation in BEAS-2B cells treated with siRNA. GR (red) with PP2A (green) translocated into the nucleus (blue) in the control [right panels in (i)], whereas GR with PP2A remained in the cytoplasm even after the addition of Dex in cells with reduced PP2A by siRNA (right panels in (ii)). The arrow heads indicate GR with PP2A in the cytoplasm. Images were obtained using a Carl Zeiss LSM700 confocal microscope (400× objective).

We further evaluated PP2A’s involvement in GR nuclear translocation during eosinophilic inflammation. When BEAS-2B airway bronchial cells were stimulated with eosinophil peroxidase (EPX), an eosinophil granule protein, PP2A became phosphorylated in a dose-dependent manner (Figure 2A). In addition, PP2A protein expression was slightly, but significantly, reduced (Figure 2B), concomitant with a 20% reduction in PP2A activity (Figure 2C). This reduction in PP2A activity was likely accounted for in terms of the degradation of PP2A by its phosphorylation, as we have shown previously [11]. Since PP2A was detected in GR-immunoprecipitates obtained from BEAS-2B cell extracts (Figure 2D), PP2A may be associated with GR function in the same complex [10]. Finally, we revealed that reduced PP2A by siRNA impaired GR’s ability to translocate to the nucleus (Figure 2E), suggesting that activated eosinophils could reduce PP2A and the response to corticosteroids at local inflammatory sites.

### 3.3. Eosinophilic Inflammatory Mediators are Increased in the Epithelial Cells of Nasal Polyps

**Figure 3:**
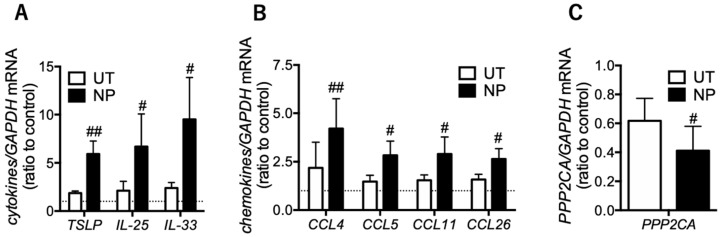
Inflammatory mediators and PP2A gene expression in epithelial cells of nasal polyps. mRNA levels of innate immune response-associated cytokines (**A**), eosinophil-recruiting chemokines (**B**), and PP2A (**C**) were determined using RT-PCR in both epithelial cells of uncinate process tissue (UT) and nasal polyps (NP) in the ethmoid sinus obtained from the ECRS patients. Values represent the ratio to control (UT from patients with allergic rhinitis) and the mean ± SEM values of four subjects: ^#^ *p* < 0.05, ^##^ *p* < 0.01 (vs. UT).

When corticosteroids work properly, they suppress a number of inflammatory genes [14]. Thus, we focused on mRNA levels of inflammatory mediators associated with eosinophilic airway inflammation at local sites as regulators of corticosteroid action. In epithelial cells of nasal polyps (eosinophil-rich areas), TSLP, IL-25, and IL-33 were higher than in those of uncinate process tissues (eosinophil-poor areas) (Figure 3A). The eosinophilic chemo-attractants CCL4, CCL5, CCL11, and CCL26 were increased in nasal polyps compared with uncinate process tissues (Figure 3B). These results indicated that, in eosinophil-rich areas, corticosteroid action was impaired concomitant with reduced PP2A (Figure 3C).

### 3.4. PP2A may Present a Promising Therapeutic Target for Tocal Corticosteroid Resistance

**Figure 4:**
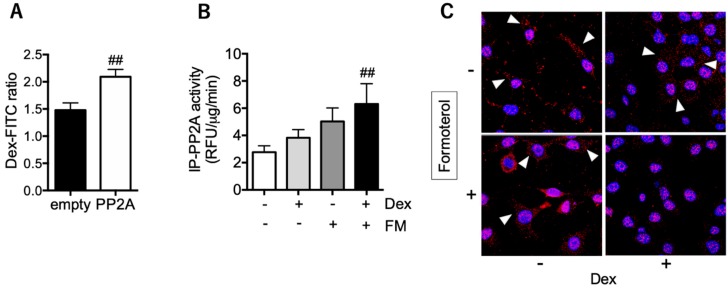
Effect of PP2A overexpression and activation on GR nuclear translocation. (**A**) The PP2A plasmid (or empty plasmid) was transfected in BEAS-2B cells. FITC-conjugated dexamethasone (Dex-FITC: 10^−6^ M overnight) in the nuclei was evaluated and represented the ratio to baseline (time = 0). (**B**,**C**) Epithelial cells of nasal polyps were pretreated with formoterol (10^−9^ M) for 20 min, followed by incubation with dexamethasone (Dex; 10^−7^ M) for 60 min. PP2A activity (**B**) and Dex-induced GR nuclear translocation (**C**) were evaluated. Values in A and B represent the mean ± SEM values of four experiments: ^##^ *p* < 0.01 (vs. empty plasmid or vehicle). Arrowheads in C indicate GR located in the cytoplasm. Images were obtained using a Carl Zeiss LSM700 confocal microscope (400× objective).

We confirmed that PP2A overexpression enhanced GR nuclear translocation in BEAS-2B cells (Figure 4A). In addition, the long-acting β_2_ adrenoceptor agonist (LABA) formoterol activated PP2A cooperation with corticosteroid (Figure 4B), resulting in improved levels of GR nuclear translocation in nasal polyp epithelial cells from patients with ECRS (Figure 4C). Taken together, PP2A may be a promising therapeutic target for reduced local responses to corticosteroids.

## 4. Discussion

In this study, we demonstrated reduced local responses to corticosteroids in ECRS patients with bronchial asthma, particularly at eosinophilic inflammatory sites (eosinophil-rich areas). Impaired PP2A in these areas resulted in reduced GR nuclear translocation and corticosteroid insensitivity. In addition, epithelial cells in these areas enhanced the production of ILC2-stimulating cytokines, which served to induce type 2 allergic responses [15] and the expression of several chemokines involved in eosinophilic inflammation [16,17,18,19]. Among these chemokines, CCL4 was found to be markedly elevated. CCL4 is also released, not only from macrophages and mononuclear cells, but also activated eosinophils [16]. Since CCR5, a specific receptor for CCL4 [20], is expressed on eosinophils, T cells, and ILC2 [21], these cells could be further recruited in eosinophil-rich areas and cooperatively enhance type 2 allergic responses.

We recently showed that eosinophils are activated in eosinophil-rich nasal polyps compared with peripheral blood eosinophils in the same patients [22]. Several granule proteins, such as EPX and EDN, degranulate from activated eosinophils [23]. In addition, our results demonstrated that PP2A phosphorylation levels were elevated in epithelial cells after EPX stimulation (Figure 2A) but not in those under co-incubation with non-activated eosinophils (Figure S1). The PP2A catalytic subunit, a regulator of PP2A complexes and activity [24], is phosphorylated at the Tyr^307^ residue [25], which reduces PP2A activity [26]. Thus, at local sites where eosinophils are activated, impaired PP2A may induce corticosteroid insensitivity. As one of the mechanisms for PP2A hyperphosphorylation in eosinophilic airway inflammation, we confirmed that PTP-RR, a regulator of PP2A-Tyr^307^ phosphorylation [11], was reduced under stimulation with EPX and in nasal epithelial cells from ECRS patients with severe asthma (Figure S2). Furthermore, PTP-RR expression in nasal epithelial cells was positively correlated with PP2A expression, suggesting that PTP-RR may regulate PP2A-dependant local responses to corticosteroids.

Regarding the therapeutic targets of local corticosteroid insensitivity, restoration of PP2A function may be a candidate. Increased PP2A expression with plasmids, or LABA’s activation of PP2A, may enhance responses to corticosteroids, as we have previously shown in mononuclear cells [10,27]. In same cases of refractory eosinophilic airway inflammation, increased drug delivery to the local inflammatory site with a novel method using inhaled ICS/LABA ETN has been shown to be effective [5]. Our findings are supported by recent reports showing that LABA ameliorates eosinophilic inflammation via PP2A activation [28,29].

Milara et al. reported that responses to corticosteroids were reduced concomitant with the downregulation of the mucin 1protein in nasal polyp epithelial cells from patients with refractory chronic rhinosinusitis [8]. Mucin 1 may contribute to corticosteroid-induced MAP kinase phosphatase 1 (MKP1) upregulation [8], inhibiting MAPK activity and GR-Ser^226^ phosphorylation, which is associated with reduced GR nuclear translocation [10]. Although the interaction between mucin 1 and PP2A has not been elucidated, we confirmed mucin 1 and PP2A are located in the same complex from GR-immunoprecipitates (Figure S3). Further study is warranted to investigate mucin 1’s role in the PP2A-dependent regulation of corticosteroid sensitivity.

## 5. Conclusions

In this study, we evaluated one of the molecular mechanisms of reduced local response to corticosteroids in ECRS with asthma and showed PP2A may present a promising therapeutic target. Our findings contribute to the management of refractory eosinophilic airway inflammation.

## Supplementary Materials

The following are available online at https://www.mdpi.com/2218-273X/10/2/326/s1, Figure S1: PP2A phosphorylation in BEAS-2B cells co-incubated with non-activated eosinophils, Figure S2: Involvement of PTP-RR in corticosteroid sensitivity, Figure S3: PP2A and mucin 1 expression in GR-immunoprecipitates, Table S1: Patients’ characteristics, Table S2: Amplification primers (5′–3′).
