# Supplementary material for: Reduced Local Response to Corticosteroids in Eosinophilic Chronic Rhinosinusitis with Asthma
Source: Biomolecules. 2020 Feb 18;10(2):326. doi: 10.3390/biom10020326 (PMC7072408; doi:10.3390/biom10020326)
Supplement: Supplementary file 1 [file biomolecules-10-00326-s001.zip › Supplementary Figures.pdf]

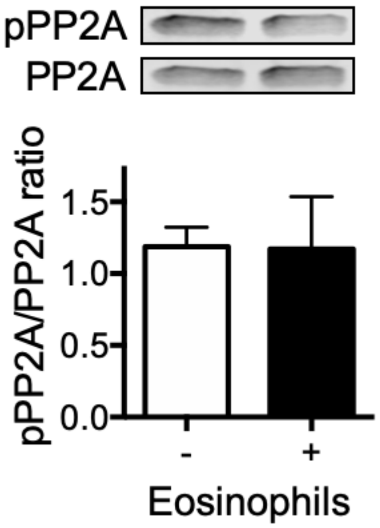

Figure S1. PP2A phosphorylation in BEAS-2B cells co-incubated with non-activated eosinophils. BEAS-2B cells were co-incubated with purified blood eosinophils for 3 days. Phosphorylation levels of PP2A were evaluated. Data are expressed as the ratio to PP2A and the values represent the mean ± SEM values of three experiments.

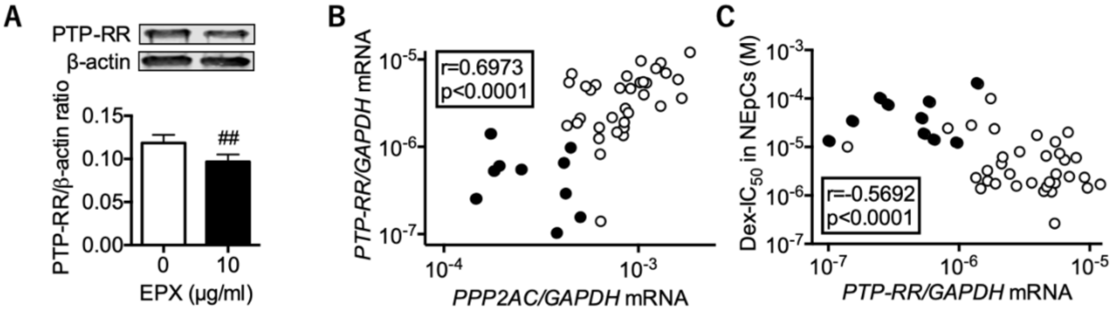

Figure S2. Involvement of PTP-RR in corticosteroid sensitivity. ***A,*** PTP-RR protein expression in BEAS-2B cells co-incubated with recombinant eosinophil peroxidase (EPX) for 3 days. Values represent the mean ± SEM values of four experiments: ##$p < 0.01$ (vs. vehicle). β-actin bands were the same bands in Figure 2B. ***B,*** Correlation between PTP-RR and PP2A mRNA levels in nasal epithelial cells (NEpCs). ***C,*** Correlation between Dex-IC_{50} and PTP-RR mRNA levels in NEpCs. The filled circles indicate individual values of ECRS patients with severe asthma.

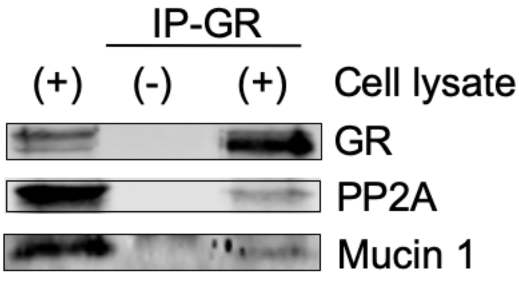

Figure S3. PP2A and mucin 1 expression in GR-immunoprecipitates. GR, PP2A and Mucin 1 were detected in whole cell extracts (left lane) and in GR-immunoprecipitates (right lane).
