# Supplementary material for: Reduced Local Response to Corticosteroids in Eosinophilic Chronic Rhinosinusitis with Asthma
Source: Biomolecules. 2020 Feb 18;10(2):326. doi: 10.3390/biom10020326 (PMC7072408; doi:10.3390/biom10020326)
Supplement: Supplementary file 1 [file biomolecules-10-00326-s001.zip › Supplementary Tables.pdf]

Table S1. Patients' characteristics

	HV (n=10)	ECRS w/o asthma (n=6)	ECRS with NSA (n=10)	ECRS with SA (n=12)	Non-ECRS (n=10)	AR (n=5)
Age	54.2 ± 12.2	55.8 ± 11.0	56.5 ± 13.5	62.5 ± 6.4	59.8 ± 15.4	34.2 ± 23.8
Gender (M/F)	3/7	4/2	4/6	7/5	9/1	4/1
Smoking status (never/ex)	9/1	2/4	4/6	6/6	4/6	4/1
Eosinophils (per μL)	186 ± 122	263 ± 212	390 ± 222	509 ± 592	227 ± 163	243 ± 196
FEV_1 (%predicted)	92.9 ± 8.6*	97.7 ± 9.2**	85.1 ± 15.4	64.3 ± 19.9	94.2 ± 13.5**	87.0 ± 10.8
FEV1/FVC	81.1 ± 5.5*	81.5 ± 3.8*	75.3 ± 7.7	64.3 ± 15.1	74.9 ± 7.7	84.4 ± 5.6**
FEF_{25-75} (%predicted)	76.1 ± 17.1**	83.3 ± 20.8**	55.3 ± 20.7	35.0 ± 17.7	63.5 ± 24.6	73.3 ± 20.3
Treatment						
ICS (μg)[1)]	none	none	690 ± 260	892 ± 312	none	none
LABA	0	0	7	12	0	0
LTRA	0	1	6	9	0	3
Anti-histamine	0	0	1	6	1	2
INS	0	4	5	10	0	1
OCS	0	0	0	3 [1.8 ± 0.6mg][2)]	0	0
Macrolide	0	1	0	2	3	0

AR: allergic rhinitis; ECRS: eosinophilic chronic rhinosinusitis; FEF_{25-75}: forced expiratory flow between 25% and 75% of vital capacity; FEV_1: forced expiratory volume in 1 second; FVC: forced vital capacity; HV: healthy volunteers; ICS: inhaled corticosteroid; INS: inhaled nasal corticosteroid; LABA: long-acting β_2-adrenergic agonist; LTRA: leukotriene receptor antagonist; NSA: non-severe asthmatics; OCS: oral corticosteroid; SA: severe asthmatics, [1)] fluticasone propionate equivalent dose, [2)] prednisolone equivalent dose, *P<0.05, **P<0.01 (vs. ECRS with SA)

Table S2. Amplification primers (5′–3′)

gene	forward	reverse
PPP2CA	CGC CAT TAC AGA GAG CCG AG	GTA CTT CTG GCG GCT GTT GA
PTP-RR	TGG TTT GGC AGG AAG ACA GC	TTT CCG GCC AGT ATA GCA CAC
TSLP	CCG CCT ATG AGC AGC CAC	CCT CAG TAG CAT TTA TCT CAG
IL-25	GAG ATA TGA GTT GGA CAG AGA CTT GAA	CCA TGT GGG AGC CTG TCT GTA
IL-33	GAA GAA CAC AGC AAG CAA AGC	TAC CAA AGG CAA AGC ACT CC
CCL4	CTC CTC ATG CTA GTA GCT GCC TTC	GGT GTA AGA AAA GCA GCA GGC GGT
CCL5	GTT GCA CCA AGC TAT GCA GGT	GCA GAA GCG TTT GGC AAT GT
CCL11	CCA CAC TGA AGG TCT CCG CA	TCT CTA GTC GCT GAA GGG GT
CCL26	ACT CCG AAA CAA TTG TGA CTC AGC TG	GTA ACT CTG GGA GGA AAC ACC CTC TCC
GAPDH	TTC ACC ACC ATG GAG AAG GC	AGG AGG CAT TGC TGA TGA TCT
